# Nutritional ketosis modulates the methylation of cancer-related genes in patients with obesity and in breast cancer cells

**DOI:** 10.1007/s13105-025-01076-9

**Published:** 2025-03-27

**Authors:** Paula M Lorenzo, Andrea G Izquierdo, Gemma Rodriguez-Carnero, Nicolas Costa-Fraga, Angel Díaz-Lagares, Cristina Porca, Daniel de Luis, Cristina Tejera, Laura De Paz, Juan Cueva, Diego Bellido, Ana B Crujeiras

**Affiliations:** 1 Epigenomics in Endocrinology and Nutrition Group, Epigenomics Unit, Instituto de Investigacion Sanitaria de Santiago de Compostela (IDIS), Unidad de Epigenomica. Complejo Hospitalario Universitario de Santiago de Compostela (CHUS/SERGAS), Travesía da Choupana Street s/n, Santiago de Compostela, La Coruña, 15706 Spain; 2 CIBER Fisiopatologia de La Obesidad y Nutricion (CIBERobn), Madrid, Spain; 3 Endocrinology and Nutrition Department, Complejo Hospitalario Universitario de Santiago de Compostela (CHUS/SERGAS), Santiago de Compostela, Spain; 4 Epigenomics Unit, Cancer Epigenomics, Translational Medical Oncology Group (ONCOMET), Instituto de Investigación Sanitaria de Santiago (IDIS), Complejo Hospitalario Universitario de Santiago de Compostela (CHUS/SERGAS), Santiago de Compostela, Spain; 5 Centro de Investigacion Biomedica en Red Cancer (CIBERONC), ISCIII, Madrid, Spain; 6 Department of Clinical Analysis, Complejo Hospitalario Universitario de Santiago de Compostela (CHUS/SERGAS), Santiago de Compostela, Spain; 7 Endocrinology and Nutrition Department, Complejo Hospitalario Universitario de Ferrol (CHUF/SERGAS), Ferrol, Spain; 8 Center of Investigation of Endocrinology and Nutrition, Medicine School, Department of Endocrinology and Investigation, Hospital Clinico Universitario, University of Valladolid, Valladolid, Spain; 9 Medical Oncology Department, Complejo Hospitalario Universitario de Ferrol (CHUF/SERGAS), Ferrol, Spain; 10 Medical Oncology Department, Complejo Hospitalario Universitario de Santiago de Compostela (CHUS/SERGAS), Santiago de Compostela, Spain

**Keywords:** Ketogenic diet, Ketone bodies, Breast cancer, Epigenetics, Adipose tissue, Tumor suppressors, Oncogenes, Sirtuins, DNMTs

## Abstract

Scientific evidence demonstrates that a very low-calorie ketogenic diet (VLCKD) is effective and beneficial in the treatment of obesity, capable of reversing the methylome associated with obesity and has immunomodulatory capacity. This effect is in part promoted by nutritional ketosis and could be involved in counteracting obesity-related cancer. The aim of this study was to evaluate the effect of nutritional ketosis on the methylation of genes related to tumor processes in patients with obesity and in breast cancer cells. Based on methylome data (Infinium MethylationEPIC BeadChip, Illumina) from patients with obesity treated with a VLCKD for weight loss (*n* = 10; *n* = 5 women, age = 48.8 ± 9.20 years, BMI = 32.9 ± 1.4 kg/m2), genes belonging to cancer-related pathways were specifically evaluated and further validated in vitro in MDA-MB-231 (triple negative) and MCF7 (RE positive) breast tumor cells pretreated for 72 h with βOHB, the main ketone body, secretome from visceral (VATs) or subcutaneous (SATs) adipose tissue of patients with obesity. The cell tumoral phenotype was evaluated by proliferation assay and expression of cancer-related genes. VLCKD-induced nutritional ketosis promoted changes in the methylation of 18 genes (20 CpGs; 17 hypomethylated, 3 hypermethylated) belonged to cancer-related pathways with MAPK10, CCN1, CTNNA2, LAMC3 and GLI2 being the most representative genes. A similar pattern was observed in the MDA-MB-231 cells treated with β-OHB, without changes in MCF7. These epigenetic changes paralleled the tumoral phenotype modulated by the treatments. Taking together these results highlight the potential role of VLCKD as an adjuvant to anticancer treatment in groups more susceptible to the development of cancer such as patients with obesity, exerting epigenetic regulation through nutritional ketosis and weight loss.

**Supplementary Information:**

The online version contains supplementary material available at 10.1007/s13105-025-01076-9.

## Introduction

Obesity, considered the pandemic of the 21st century, in addition to being a disease in itself, is also a powerful risk factor for numerous diseases, including cancer, with breast cancer among the most affected. Despite consistent current epidemiological evidence, the molecular mechanisms underlying the association between obesity and increased risk of cancer incidence are still unclear [1]. Among the proposed mechanisms, the secreted factors yielded by the visceral adipose tissue (VAT) dysfunction in obesity that lead to a state of chronic low-grade inflammation and oxidative stress in the body is currently the most studied [2]. It was hypothesized that the persistent inflammation associated to obesity creates a favorable microenvironment for uncontrolled cell proliferation and is related to different phases of tumor development [3]. The proinflammatory and prooxidant factors can affect the expression levels and activity of enzymes involved in epigenetic regulation, leading to alterations in DNA methylation (either hypermethylation or hypomethylation), posttranslational histone modifications (resulting in active or repressive marks), and the expression of noncoding RNAs, such as microRNAs. These epigenetic disruptions may impact the function of tumor suppressor genes and oncogenes, potentially initiating the carcinogenesis process [4]. In fact, these molecular mechanisms have been linked to the promotion of several types of cancer, including breast cancer [5, 6], where women with obesity have been observed to have elevated levels of inflammatory and oxidative stress markers, correlated with a worse prognosis and greater tumor aggressiveness. The effect of these obese-related microenvironment on carcionogenesis could be mediated by epigenetic mechanisms so that inflammation and oxidative stress, characteristics of obesity, and epigenetic marks have been proposed as mechanistic links in the association of excess abdominal adiposity and the development of carcinogenesis [4, 7, 8].

It was estimated that between 30% and 40% of cancers could be prevented through lifestyle changes, such as improving diet, promoting physical activity and less exposure to environmental factors, showing that weight loss interventions could be a good strategy to counteract the risk and poor prognosis of obesity-related cancer [9]. In this context, scientific evidence demonstrates that a very low-calorie ketogenic diet (VLCKD) is effective and beneficial in the treatment of obesity, not only about effective weight loss and preservation of muscle mass [10], but it also has the ability to change the obesity-associated methylome in a way more similar to the methylome observed in normal-weight volunteers [11] and has immunomodulatory capacity [12].

Recently, the ketogenic diet has been proposed as an adjuvant therapy in cancer treatment [13–16]. This proposal was focused on the fact that it reduces circulating glucose levels and induces ketosis, thereby depriving cancer cells of energy while normal cells adapt their metabolism to use ketone bodies as fuel to survive. Furthermore, lowering blood glucose levels also decreases levels of insulin and insulin-like growth factors, which are known to be relevant drivers of cancer cell proliferation [13]. This effect of nutritional ketosis could be in part promoted by epigenetic mechanisms [17]. Ketone bodies act not only as alternative sources of energy, but also as signaling molecules capable of modulating the epigenetic structure of cells [18, 19]. In the context of the ketogenic diet, these epigenetic changes may collaborate with existing anticancer therapies by making tumor cells more susceptible to treatments such as chemotherapy and radiotherapy [15]. The ketogenic diet affects the levels of metabolic intermediates such as acetyl-CoA and S-adenosylmethionine (SAMe), which are crucial for epigenetic modifications. Changes in the availability of these metabolites can influence DNA and histone methylation patterns, favoring the expression of cancer-fighting genes. In addition, β-OHB can reduce oxidative stress and inflammation, both of which are associated with cancer-promoting epigenetic changes. This may result in more favorable regulation of the epigenome, promoting a less permissive environment for tumor growth [15].

The reversibility of epigenetic marks is one of the most important and prominent features of epigenetic regulation, mainly in DNA methylation. These epigenetic modifications induced by the environment or lifestyle offer relevant information to understand the increase or decrease in susceptibility to cancer [20]. Therefore, nutrients, diet [21] or physical exercise [22] can also directly influence DNA methylation. The presence or absence of certain nutrients and bioactive compounds in the diet is related to epigenetic modifications in genes that regulate metabolic processes, and these modifications can predispose to the development of metabolic disorders and some types of cancer [21].

The aim of this study was to evaluate the effect of nutritional ketosis on the methylation of genes related to tumor processes in patients with obesity and if this effect can be detected in breast cancer cells in association with their tumoral phenotype measured by the proliferation and the expression of genes involved in the early-steps of carcinogenesis.

## Materials and methods

### Patients

The study population included 10 participants (5 men/5 women) with an average age of 48.8 ± 9.2 years and an average body mass index (BMI) of 32.9 ± 1.4 Kg/m^2^ undergoing a nutritional intervention based on a very low-calorie ketogenic diet (VLCKD), recruited in collaboration with the Endocrinology and Nutrition Department of the University Clinical Hospital of Valladolid, Spain. This intervention had a maximum duration of 6 months and the analyses were carried out from the samples collected at 0 (Baseline) and 1–2 months (Maximum Ketosis) from the beginning of the treatment for weight loss.

The VLCKD is a nutritional intervention was based on a commercial weight-loss program (PNK method ^®^), as described elsewhere [10]. The weight-loss program has five steps and adheres to the most recent guidelines of the EFSA (2015) on total carbohydrate intake [23]. The first three steps are a ketogenic phase of this method that consist of a VLCKD (600–800 kcal/day) that is low in carbohydrates (< 50 g daily from vegetables) and lipids (only 10 g of olive oil per day). Throughout these ketogenic phases, supplements of vitamins and minerals, such as K, Na, Mg, Ca, and omega-3 fatty acids, were provided in accordance with international recommendations. These three steps are maintained until the patient lost the target amount of weight, ideally 80%. Because of this, the ketogenic steps varied in time depending on the individual and the weight-loss target. The total ketosis state lasted for a maximum of 60 days.

The study protocols from which the data were collected were performed in accordance with the Declaration of Helsinki and was approved by the Ethics Committee for Clinical Research of Hospital Clinico Universitario de Valladolid, Spain (C.I:40/13). Participants provided written informed consent before any intervention related to the study. Participants received no monetary incentives.

### Cell lines

The human breast cancer cells, MCF-7 and MDA-MB-231 were provided by American Type Culture Collection (ATCC) (HTB-22, ATCC, Virginia, EE.UU.). The MCF7 and MDA-MB-231 cells were maintained in Dulbecco’s modified Eagle medium (DMEM) (Lonza, Iberica S.A. Barcelona, Spain) supplemented with 10% Fetal Bovine Serum (FBS) (Lonza, Iberica S.A. Barcelona, Spain), and 1% penicillin-streptomycin (Lonza) in 5% CO2 humidified atmosphere at 37˚C.

### Cell lines treatments.

#### Ketone bodies

MCF7 and MDA-MB-231 cell lines were grown in their usual culture medium with sodium salt of β-OHB (Sodium 3-hydroxybutyrate, Sigma-Aldrich; Merck KGaA, Darmstadt, Germany) diluted (1 M stock concentration, H2O diluent treated with diethylpyrocarbonate (DEPC)) for 24–72 h, renewing the medium every 24 h. After exposure to the sodium salt of β-OHB, the cells were collected as cell lysates and stored at -80ºC for RNA and DNA isolations.

#### Adipose tissue secretomes

MCF7 and MDA-MB-231 cells were subjected to treatments with VAT or SAT secretomes. Samples from which these treatments were developed come from patients with severe obesity, extracted at the time of undergoing bariatric surgery for weight loss. The treatments were prepared from the pool of three samples from the different aliquots obtained from patients with obesity, at a tested concentration of 1% or 5% (v/v), selected according to previous studies [5]. Cells were treated for 24–72 h, renewing the medium with secretomes every 24 h. After treatment with secretomes, the cells were collected as cell lysates and were stored at -80ºC for RNA and DNA extractions.

### Cell proliferation assay

Proliferation of MCF7 and MDA-MB-231 cells was estimated using the Cell Proliferation Reagent WST1 (Roche, Basel, Switzerland). Briefly, 5 × 10^3^ MCF7 or MDA-MB-231 cells were seeded into a 96-well plate and incubated overnight for attachment. The complete medium was then replaced with serum-free medium to allow for cell cycle synchronization, and treatment was added after 24 h. The cells were incubated with VAT or SAT secretomes from patients with severe obesity at 1% and 5% concentration as previously described [5], or with a range of 10–200 mM of β-OHB for 24, 48 and 72 h. Specific cell line serum-free culture medium was considered as a control. The treatments were performed in quadruplicate in at least three independent experiments and normalized against the control.

### Gene expression assessment

RNA was isolated from the cell lines using the commercial kit GeneJet RNA Purification Kit (ThermoScientific, Waltan, MA, EE. UU.) according to the manufacturer’s recommendations. RNA concentrations and purity were measured with NanoDrop 2000c equipment (Thermo Scientific). Extracted total RNA was purified with DNase treatment using a DNA-free kit as a template (Ambion) to generate first-strand cDNA synthesis using the High-Capacity cDNA Reverse Transcription Kit (Applied Biosystems). The expression of genes was assessed using TaqMan real-time PCR and a Step One Plus system (Applied Biosystems, USA) with specific primers and probes for the genes that were obtained from inventoried TaqMan Gene Expression Assays (Applied Biosystems, USA). All reactions were performed using the following cycling parameters: 50 °C for 2 min, 95 °C for 10 min, followed by 40 cycles of 95 °C for 15 s, 60 °C for 1 min. All experiments were performed in duplicate and gene expression levels were normalized using *GAPDH* as an internal control. The fold change in gene expression was calculated using the 2^−ΔΔCt^ relative quantitation method according to the manufacturer’s guidelines (Applied Biosystems), and data are reported as mean ± standard error of the mean (SEM). RT-qPCR experiments were performed in compliance with the MIQE (Minimum Information for Publication of Quantitative Real-Time PCR Experiments) guidelines (http://www.rdml.org/miqe). The commercially available and pre-validated TaqMan primer/probe sets used are shown in the Supplementary Table 1.

### DNA methylation analysis

The DNA methylation profile of the studied genes was evaluated by analysing a methylome dataset of blood leukocytes samples from patients with obesity undergoing a VLCKD, and of samples derived from breast cell lines.

Data from the MethylationEPIC v1.0 BeadChip (Illumina) methylomes of blood leukocytes were obtained at time points of ketotic phases as previously published [11].

DNA from breast cancer cell lines samples (MDA-MB-231 and MCF7) were isolated using a standard phenol-chloroform/proteinase-k protocol according to the manufacturer’s instructions, with slight modifications. The isolated DNA was treated with RNase A for 1 h at 45 °C. All DNA samples were quantified using the fluorometric method (Quan-iT PicoGreen DsDNA Assay, Life Technologies) and were assessed for purity and quality using a spectrophotometer (NanoDrop 2000c, ThermoScientific) to determine 260/280 and 260/230 ratio measurements. High-quality DNA samples were selected for bisulfite conversion (500 ng) using a commercial kit (DNA Methylation™ Kit, Zymo Research) according to the manufacturer’s instructions, which converts unmethylated cytosines to uracil.

Methylome data were obtained through hybridization of the samples on the Infinium MethylationEPIC v1.0 BeadChip (Illumina), following the Illumina Infinium HD methylation protocol, and analysis was performed on a HiScan SQ module (Illumina) to evaluate cytosine methylation states. Image intensities were extracted using GenomeStudio (V2010.3) Methylation Module (1.9.0) software from Illumina. The annotation of CG islands (CGIs) used the following categorization: (1) “shore” for each of the 2 kb sequences flanking a CGI; (2) “shelf” for each of the 2 kb sequences adjacent to a “shore”; and (3) “open sea” for DNA not included in any of the previous sequences or in CGIs. The transcription start site 200 and transcription start site 1500 refer to regions located 200–1500 bp, respectively, from the transcription start site.

For all samples, data quality control was assessed with GenomeStudio Illumina software (V2010.3) and BeadArray Controls Reporter (V1.1) from Illumina, based on the internal control probes present on the array. The methylation score of each CpG from samples that passed this quality control was represented as the β-value and previously normalized for color bias and background level adjustment and quantile normalization across arrays. β-values were obtained as the ratio of the fluorescent signal of the methylated (M) probe relative to the sum of the M and unmethylated (U) probes (β = M/(M + U)). The β-values range from 0 (no methylation) to 1 (completely methylated). Probes and sample filtering involved a two-step process for removing SNPs and unreliable β-values with a high detection p value > 0.01. After this filtering step, the remaining CpGs were considered valid for the study. Additionally, for in vitro assays, methylation data were processed in R (v4.2.0) using Bioconductor packages for high-throughput genomic analysis and preprocessing and quality control were performed with the minfi package.

### Statistical analysis

The sample size of the current study was calculated to detect differences in methylation levels ≥ 2%, α = 0.05 and a power (1 − β) of 80% and taking into account published values of epigenome-wide analysis in the field of obesity [24, 25]. Under these conditions and performing a two-side paired student’s t test, a sample size of at least 9 participants was calculated for the total sample size.

As indicated in the previous published study [11], a linear model was fitted using a B-spline approximation to identify consistent patterns of DMCpGs due to the nutritional intervention. P values in the human study were adjusted for multiple comparisons using the false discovery rate (FDR) procedure of Benjamini and Hochberg, and results were considered statistically significant when FDR < 0.10. Additionally, we applied a threshold for the significant sites based on the mean difference between visits with a minimum β value change of ± 0.02.

In the current study, first, genes belonging to cancer-related pathways, were specifically evaluated based on a previous Gene Ontology (GO) analysis of methylome data from patients with obesity treated with VLCKD for weight loss [11]. In the Gene Ontology (GO) enrichment analysis, to determine which entries were associated with cancer, the following approach was used: The enriched GO terms were manually reviewed, and those with an adjusted p-value < 0.05 and directly related to cancer were selected. Specifically, in our study, the chosen GO terms were those identified as “Item Kegg:05200 - Pathways in cancer.” The focus was therefore on the terms most directly associated with cancer, which included the list of 18 analysed genes (MMP9, HRAS, CHUK, ERBB2, LAMA2, IGF1R, CCND1, TGFB3, WNT2, MAPK10, CRK, LAMC1, CTNNA2, LAMC3, WNT3, HDAC1, GLI2, LAMB1). GO term analysis of our previous published study uncovered pathways with a greater than expected number of differentially methylated genes (FDR < 0.05). For a more comprehensive analysis, a STRING analysis was performed to obtain the gene-protein interaction network of these genes and the most relevant ones were identified. Then, the CpG sites of the identified genes were filtered using the Genome Studio Illumina software (V2010.3) and β values were compared between baseline and maximum ketosis by means of a paired Student’s t-test. A heatmap was performed with the filtered CpG sites that showed the highest differences in methylation leves (> 4%).

In the in vitro assays, differential methylation analysis between groups was carried out with the limma package, applying linear models to identify differentially methylated CpGs with p-values < 0.05 and absolute methylation differences (Δβ) exceeding ± 0.02. For this analysis the FDR was not statistically significant and then the P-value was considered. For the analysis of differences between groups in the proliferation and gene expression, samples from treated cells were compared with the control cells by means of Student’s t-test.

Statistical analyses were performed using SPSS version 25.0 software (SPSS Inc., Chicago, IL) for Windows 10 (Microsoft, Redmond, WA) and SPSS version 28.0 (SPSS Inc., Chicago, IL) for macOS High Sierra (Apple, EE.UU.) and the R statistical environment (version 4.2.0). *P* ≤ 0.05 was considered statistically significant.

## Results

### Effect of nutritional ketosis induced by a VLCKD on the methylation of genes related to cancer in leukocytes from patients with obesity

**Fig. 1:**
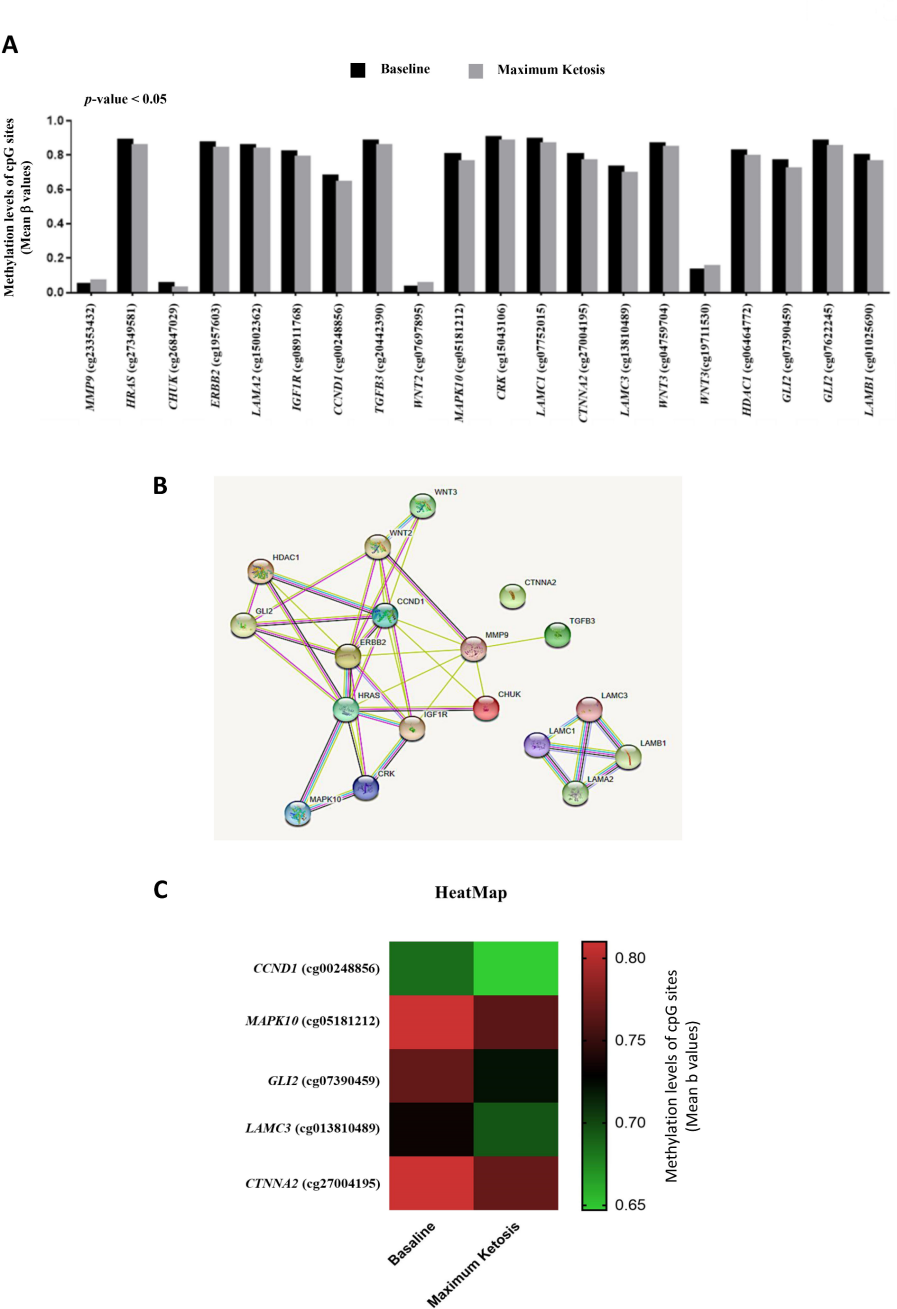
Analysis of cancer-related genes in methylome associated with a very low-calorie ketogenic diet (VLCKD). **(A)** Methylation changes in cancer-related genes induced by nutritional ketosis on a VLCKD. *P*-value was calculated by means of paired Student’s t-test respect to baseline. **(B)** Network enriched in protein interactions of cancer-related genes regulated by methylation. **(C)** Supervised clustering of the CpGs that were found to be differentially methylated between baseline and maximum ketosis with changes > 4%. *MMP9*, gene encoding matrix metalloproteinase 9; *HRAS*, gene encoding the H-Ras protein; *CHUK*, conserved helix-loop-helix ubiquitous kinase gene; *ERBB2*, gene encoding the receptor tyrosine kinase; *LAMA2*, gene encoding the laminin alpha 2 subunit; *IGF1R*, gene encoding the factor receptor insulin-like growth 1; *CCND1*, gene encoding the cyclin D1 protein; *TGFB3*, gene that encodes transforming growth factor beta 3; *WNT2*, WNT family member 2 gene; *MAPK10*, gene encoding mitogen-activated protein kinase 10; *CRK*, proto-oncogene CRK, adapter protein; *LAMC1*, gene encoding the gamma 1 subunit of laminin; *CTNNA2*, gene encoding alpha 2 catenin; *LAMC3*, gene encoding the gamma 3 subunit of laminin; *WNT3*, gene encoding WNT family member 3; *HDAC1*, gene encoding histone deacetylase 1; *GLI2*, glioma-associated oncogene 2; *LAMB1*, gene encoding laminin beta 1 subunit

In order to evaluate the effect of nutritional ketosis on the epigenetic regulation of cancer-related genes in patients with obesity, a reanalysis of the methylome associated with VLCKD treatment previously published by our research group [11] was performed. The GO analysis obtained from the 1239 differentially methylated CpGs sites (920 unique genes) in the phase of maximum ketosis, showed that 18 genes (20 CpGs; 17 hypomethylated, 3 hypermethylated) belonged to pathways related to cancer (Fig. 1A). The STRING analysis yielded that 77% of these 18 genes differentially methylated during ketosis belonged to a network significantly enriched in protein interactions (*p* < 0.001), with the gene encoding mitogen-activated protein kinase 10 (*MAPK10*) showing the highest number of protein interactions (Fig. 1B). Other important genes in this network that also presented a large number of interactions were the gene that encodes the receptor tyrosine kinase (*ERBB2*), the gene that encodes the cyclin D1 protein (*CCND1*), the gene that encodes the H-Ras protein (*HRAS*) and gene encoding histone deacetylase 1 (*HDAC1*) (Fig. 1B). An independent enriched network was also observed between laminins, the gene encoding the laminin gamma 1 subunit (*LAMC1*), the gene encoding the laminin alpha 2 subunit (*LAMA2*), the gene encoding the gamma subunit laminin 3 (*LAMC3*) and the gene encoding laminin beta 1 subunit (*LAMB1*) (Fig. 1B).

A further analysis evidenced that 5 of these genes had the highest methylation changes (> 4%). These genes were *CCND1*, *MAPK10*, glioma-associated oncogene 2 (*GLI2*), *LAMC3*, and the gene encoding alpha catenin 2 (*CTNNA2*) (Fig. 1C).

### Study of the effect of in vitro treatment with ketone bodies or with secretome from adipose tissue on the methylation of cancer-related genes in breast cancer cell lines

#### Effect of ketone body or secretome from adipose tissue of patients with obesity on carcinogenesis phenotype of breast cell lines

To evaluate the effect of obesity and nutritional ketosis on carcinogenesis, an in vitro study was performed in the mammary human tumor cell lines MCF7 and MDA-MB-231. These cell lines were exposed to human AT secretomes from patients with obesity at 5% or different concentrations of β-OHB, the main ketone body of VLCKD-induced nutritional ketosis. The carcinogenesis phenotype was evaluated by the proliferation assessment and the analysis of gene expression of representative genes involved in carcinogenesis such as tumor suppressor genes (*BRCA1*, *PTEN* and *TP53*) and genes related to antioxidant protection (*SIRT1*, *SIRT3*, *SIRT6* and *GSTM2*) as tumor-protecting genes and a group of tumor-promoting genes (*BIRC5*, *MYC* and *ALDH3A1*). These genes were selected because they are representative of genes involved in early-steps of carcinogenesis and their expression was found to be modulated in the context of obesity-related features as it was showed in previous studies from our research group [5, 26]. In addition, the expression of *DNMTs* (*DNMT1*, *DNMT3a* and *DNMT3b*) was quantified because they encoded the main enzymes involved in the methylation of DNA and previously we have detected a regulation of these genes after following a VLCKD in blood leukocytes of patients with obesity [11].

##### Study of carcinogenesis phenotype in epithelial breast cell lines exposed to treatment with β-OHB

The exposure to β-OHB induced a statistically significant decrease in proliferation of the tumoral cell lines MDA-MB-231 and MCF7, from the concentration of 50 mM and highest. The effect of β-OHB was doses-dependent since the 24 h of treatment (Supplemental Fig. 1SA and B).

**Fig. 2:**
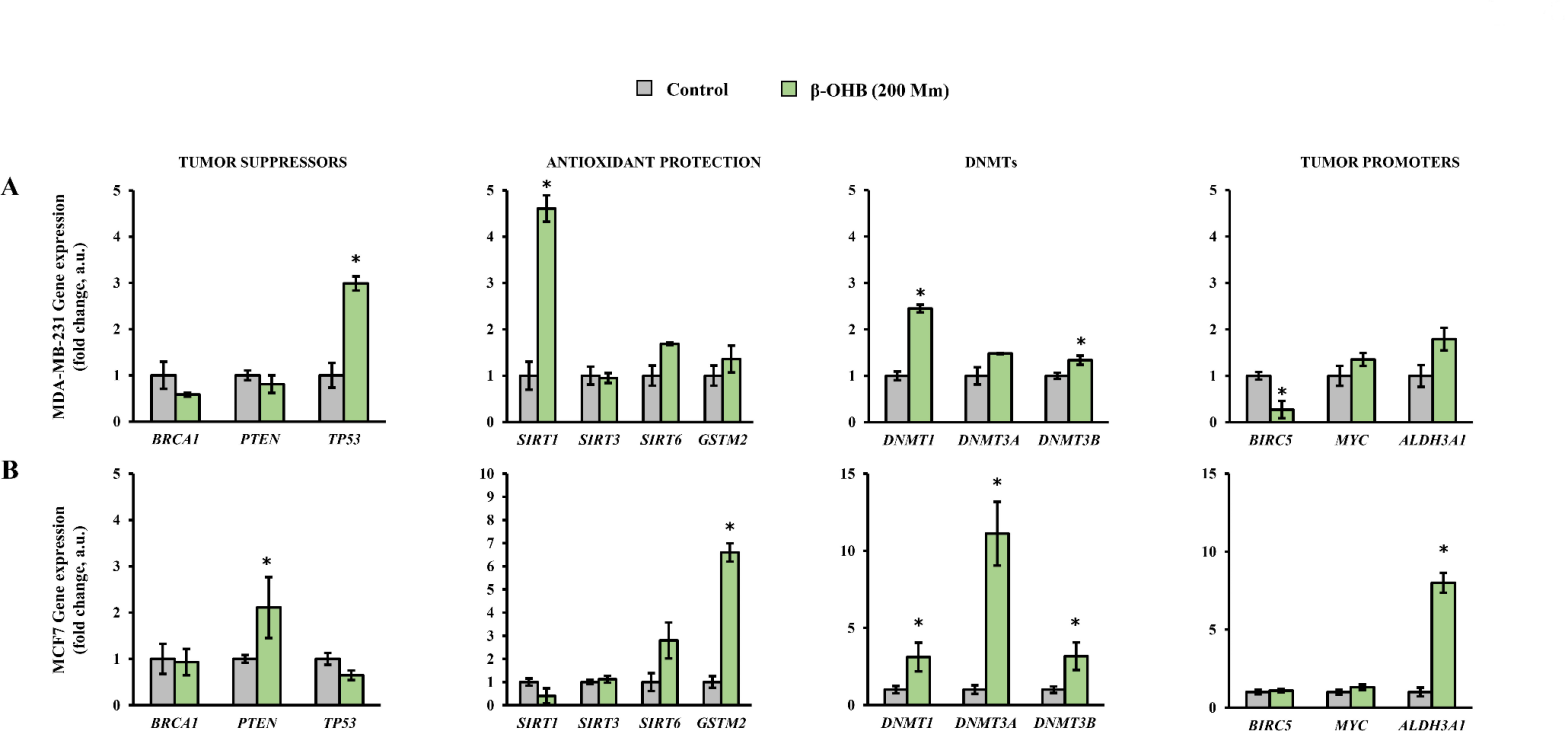
Gene expression levels of cell lines exposed to β-hydroxybutyrate (β-OHB). (**A**) MDA-MB-231 and (**B**) MCF7. Asterisk (*) denotes statistically significant differences (*p* < 0.05) compared to the control evaluated by Student’s *t*-test. BRCA1, breast cancer 1; PTEN, homolog of phosphatase and tensin; TP53, tumor protein 53; SIRT, sirtuin; GSTM2, glutathione S-transferase Mu 2; DNMT, DNA methyltransferase; BIRC5, baculoviral inhibitor of apoptosis repeat-containing 5; MYC, myelocytomatosis; ALDH3A1, aldehyde dehydrogenase 3A1

Following the cell proliferation results of cell proliferation, β-OHB concentration of 200 mM in MCF7 and MDA-MB-231 to evaluate its effect on the expression of carcinogenesis-related genes (Fig. 2) and compared with the untreated cells as control. Under these conditions differential expression was observed with a general trend to increase in tumor-protection genes and DNMTs, together to a decrease in most of the tumor-promoter genes in the two breast cancer cell lines. More specifically, in the MDA-MB-231 (Fig. 2A), a statistically significant increase in the genes *TP53*, *SIRT1*, *DNMT1* and *DNMT3B*, and a statistically significant decrease in expression of *BIRC5* was observed. MCF7 cell lines showed a statistically significant increase in the expression of *PTEN*, *GSTM2*, *DNMT1*, *DNMT3A*, *DNMT3B* and also in *ALDH3A1* (Fig. 2B).

##### Study of carcinogenesis phenotype in breast cancer cell lines exposed to human adipose tissue secretomes

**Fig. 3:**
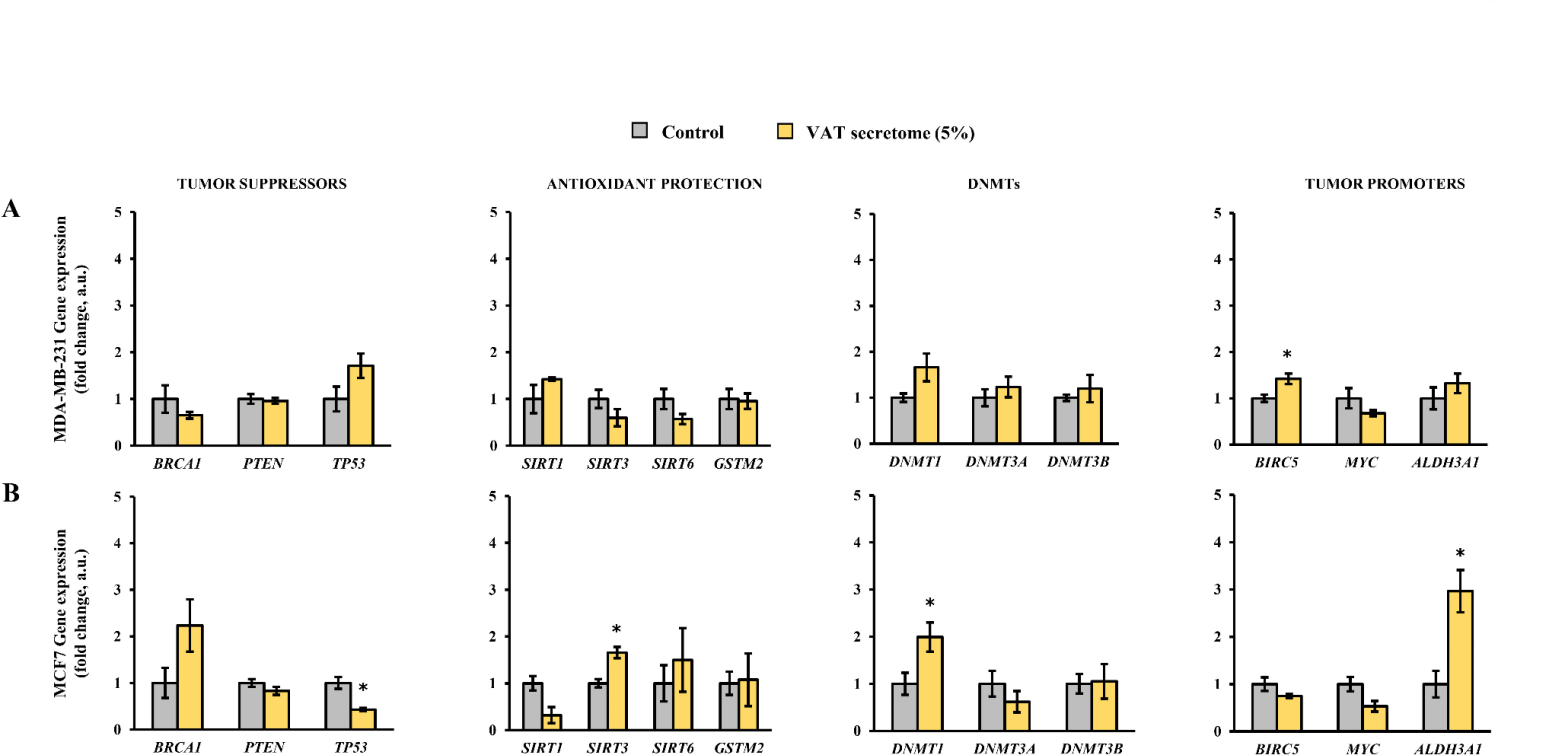
Gene expression levels of cell lines exposed to visceral adipose tissue (VAT) secretome. **(A)** MDA-MB-231 and **(B)** MCF7. Asterisk (*) denotes statistically significant differences (*p* < 0.05) compared to the control evaluated by Student’s *t*-test. BRCA1, breast cancer 1; PTEN, homolog of phosphatase and tensin; TP53, tumor protein 53; SIRT, sirtuin; GSTM2, glutathione S-transferase Mu 2; DNMT, DNA methyltransferase; BIRC5, baculoviral inhibitor of apoptosis repeat-containing 5; MYC, myelocytomatosis; ALDH3A1, aldehyde dehydrogenase 3A1

**Fig. 4:**
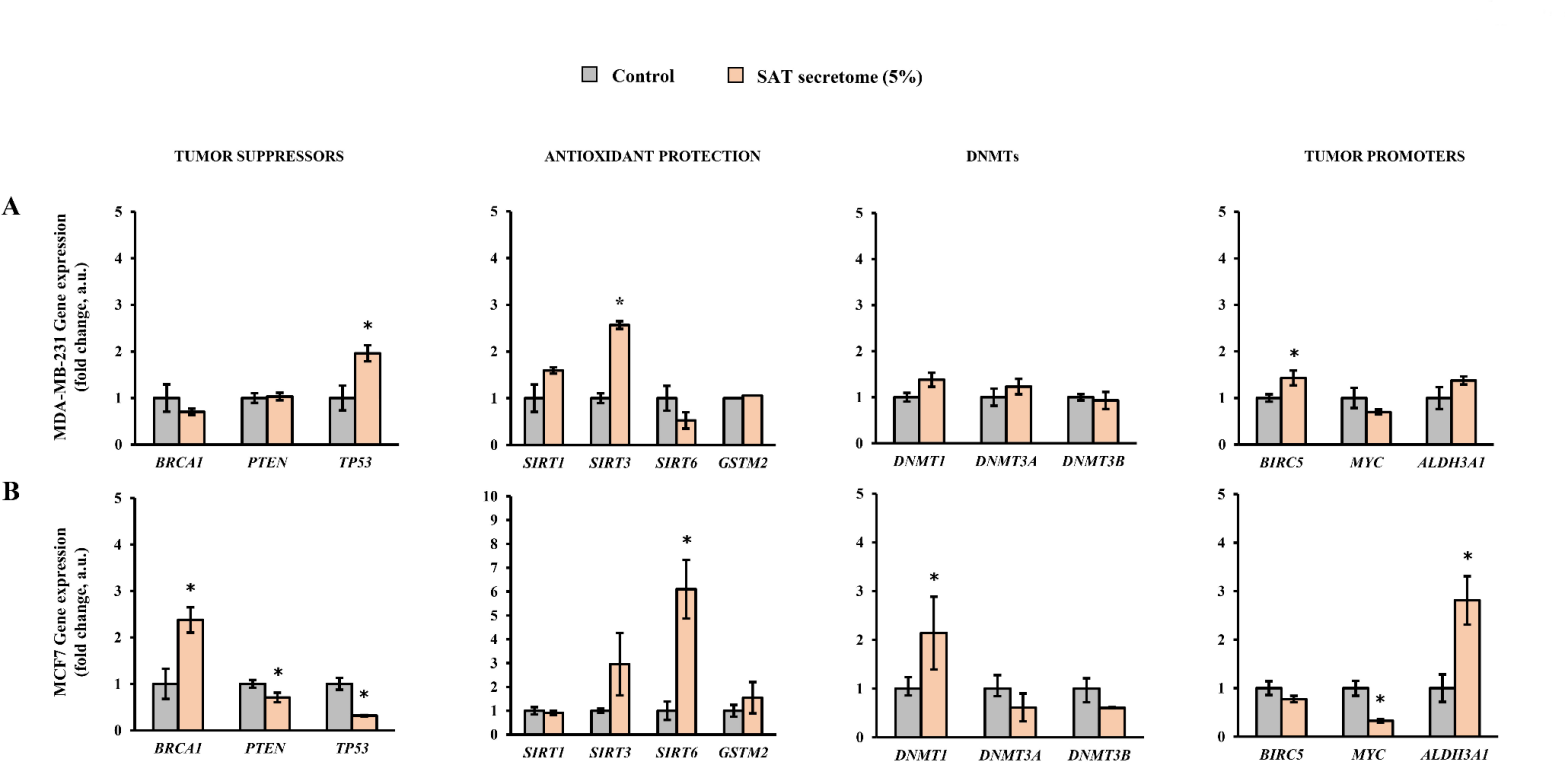
Gene expression levels of cell lines exposed to subcutaneous adipose tissue (SAT) secretome. (**A**) MDA-MB-231 and (**B**) MCF7 Asterisk (*) denotes statistically significant differences (*p* < 0.05) compared to the control evaluated by Student’s *t*-test. BRCA1, breast cancer 1; PTEN, homolog of phosphatase and tensin; TP53, tumor protein 53; SIRT, sirtuin; GSTM2, glutathione S-transferase Mu 2; DNMT, DNA methyltransferase; BIRC5, baculoviral inhibitor of apoptosis repeat-containing 5; MYC, myelocytomatosis; ALDH3A1, aldehyde dehydrogenase 3A1

To analyze the effect of obesity microenvironment, the breast cancer cell lines were exposed to human VAT or SAT secretomes from patients with obesity. The effect of VAT and SAT secretomes on the cellular proliferation was previously assessed in MCF10A [5]. Regarding to MDA-MB-231 and MCF7, a statistically significant increase was observed in both cell lines, in MCF7 this significant increase occurred after 72 h of exposure to 5% of VAT and SAT secretome, and in MDA-MB -231 the increase was observed at 48 h of treatment with 5% of VAT and SAT secretome (Supplemental Fig. 1S). Considering the proliferation assay results, VAT and SAT secretomes at 5% concentration during 72 h were used to evaluate their effect on the expression of the cancer-related genes in the breast cancer cell lines (Figs. 3 and 4). Under these conditions, a contrary pattern compared to that observed after β−OHB treatment was induced after treatment with obese-related AT secretomes in most of the studied genes. Particularly, in MDA-MB-231 exposed to VAT secretomes, a statistically significant increase in the *BIRC5* gene was observed (Fig. 3A). Regarding MCF7 exposed to VAT secretomes, a statistically significant increase in the expression of the genes *SIRT1*, *DNMT1*, and *ALDH3A1*, and a decrease in expression in *TP53* was observed. (Fig. 3B), compared to the untreated cells as control.

Regarding to the effect of SAT secretomes (Fig. 4), in MDA-MB-231 exposed to SAT secretomes, a statistically significant increase in *TP53*, *SIRT3* and *BIRC5* was observed, compared to untreated cells (Fig. 4A). MCF7 exposed to SAT secretomes showed a statistically significant increase in the expression of the genes *BRCA1*, *SIRT6*, *DNMT1*, and *ALDH3A1*, and a decrease in the expression in *PTEN*, *TP53* and *MYC* (Fig. 4B).

### Epigenetic effect of ketone bodies and obesity-related adipose tissue secretomes in breast cancer cell lines

In this study, the breast cancer cell lines, MDA-MB-231 and MCF7, were used, in which the effect of ketone bodies on the tumor phenotype had been evaluated. Both cell lines were treated for 72 h with β-OHB at a concentration of 200 mM. The DNA methylome of cells treated with β-OHB or treated with VAT or SAT secretome from patients with obesity at a concentration of 5% were compared with untreated cells by using EPIC arrays. From this analysis, methylation data (β values) of cancer-related genes previously identified in the DNA of leukocytes from patients with obesity after following a VLCKD to lose weight [11] were isolated (*CCND1*, *MAPK10*, *GLI2*, *LAMC3* and *CTNNA2*).

After the quality control of the EPIC array methylation data (β values) we were able to identify an important number of significant differentially methylated CpGs (DMCpGs) (p value < 0.05) with a difference of methylation (Δβ-value) greater than 0.02 (Δβ-value >|0.02|) between untreated and treated cancer cells (Supplementary Fig. 2A). In particular, the treatment with β-OHB yield the higher number of DMCpGs in both MCF7 (291.677 DMCpGs encoding 194.009 genes, 99% of DMCpGs gained methylation) and MDA-MB-231 lines (152.299 DMCpGs encoding 109.009 genes, 2% of DMCpGs gained methylation). Regarding to the effect of treatments with AT secretomes, VAT secretome induced highest DMCpGs than SAT in both lines. According to their CpG context and gene region location, most of the DMCpGs were located at open sea and promoter or body depending on the treatment in both lines (Supplementary Fig. 2B).

**Fig. 5:**
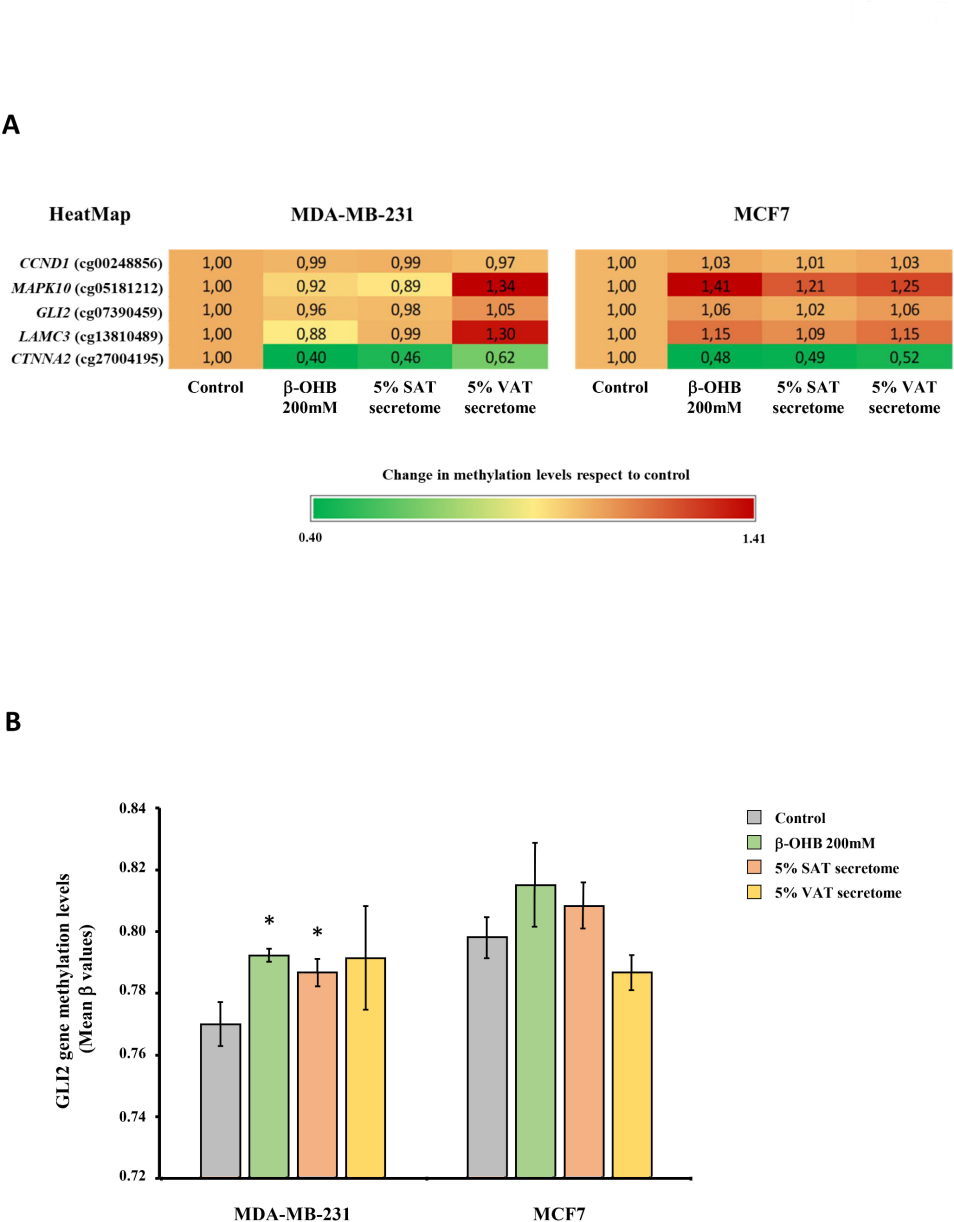
Methylation pattern in breast cancer cell lines of the cancer-related genes previously identified in patients with obesity treated with a VLCKD. **(A)** Methylation pattern induced by in vitro treatment with β-hydroxybutyrate (β-OHB) or SAT or VAT secretome in breast cancer cell lines. The data represent the changes in methylation β values in the treated cells compared to the untreated control cells. **(B)** Methylation levels of GLI2 gene in treated MDA-MB-231 and MCF7 tumor cells. The data are presented as the mean; error bars represent standard error. (*) Denotes differences statistically significant (*p* < 0.05) in relation to the control evaluated using the U Mann-Whitney test. *CCND1*, gene encoding the cyclin D1 protein; *MAPK10*, gene that encodes the protein mitogen-activated kinase 10; *GLI2*, glioma-associated oncogene 2; *LAMC3*, gene encoding the subunit laminin gamma 3; *CTNNA2*, gene that encodes alpha 2 catenin

When the targeted CpGs were particularly evaluated, the methylation pattern of these DMCpGs in MDA-MB-231 cells treated with β-OHB respect to untreated cells was similar to that observed in the human study, while the MCF7 cell line presented an inverse pattern with respect to that observed in MDA-MB-231 or human blood leukocytes (Fig. 5A). This pattern was particularly detected in the CpG site associated with the *MAPK10* gene, which presented the greatest methylation difference and with the greatest number of gene-gene interrelationships, in patients. Similar to what was observed in patients, MDA-MB-231 cells presented lower methylation levels compared to the control when treated with β-OHB and also those treated with secretome from SAT. However, MDA-MB-231 cells treated with VAT secretome presented higher methylation levels than control cells for this gene. The CpG site associated with the *LAMC3* gene presented a methylation pattern similar to *MAPK10* in these cells. However, in MCF7 cells, an increase in methylation was observed when they were treated with both β-OHB and the secretomes of both fatty depots (Fig. 5A).

A further analysis of genome-wide DNA methylation patterns from treated breast cancer cell lines showed a total of 507 CpG sites belonging to the 5 genes previously identified in blood leukocytes from patients underwent VLCKD, of which 83 were located in the promoter of target genes. The statistical analysis showed statistically significant differences compared to the untreated control in several of the CpG sites analyzed. These differences occurred with a different pattern depending on the type of cell line and also depending on the treatment (Fig. 5A).

Relevantly, when the average levels of the CpG sites located in the promoter of each of the genes were analyzed, a statistically significant change in the *GLI2* oncogene was revealed (Fig. 5B), showing higher methylation levels compared to the control, when MDA-MB-231 cells were treated with β-OHB and after treatment with SAT secretome. However, no statistically significant differences were observed in the mean methylation levels of *GLI2* after treatments in the MCF7 cell line.

## Discussion

The current study evidences that a VLCKD in patients with obesity promotes changes in the DNA methylation pattern of genes involved in cancer-related pathways. This effect could be promoted by the nutritional ketosis induced by the VLCKD. In fact, the in vitro approach of this study demonstrated a protective effect of the ketone body β-OHB on the tumoral phenotype of epithelial non-tumoral and tumoral breast cell lines, contrary to that observed when these cell lines were treated with secretomes of adipose tissue from patients with obesity. Interestingly, the treated cell lines showed a similar pattern of DNA methylation in cancer-related genes previously identified in human leukocytes after following a VLCKD. These results agree with the proposal of nutritional ketosis as a useful agent to complement the strategies in the prevention and treatment of cancer in humans [27–29]. Moreover, it was previously evidenced that a ketogenic diet low in carbohydrates and in fat content promotes beneficial effects on obesity-related features such as a reduction in visceral fat mass [30] and inflammatory biomarkers [12]. Both are factors closely involved in the association between obesity and cancer and this effect could be mediated by epigenetic mechanisms [31, 32]. Therefore, considering these results and due to the effectiveness of VLCKD also shown in weight loss in patients with obesity [10, 33, 34] makes this nutritional intervention potentially effective in breast cancer associated with obesity. Relevantly, the in vitro findings of this study highlight that the interplay between the type of secretome (VAT vs. SAT) and the intrinsic characteristics of the cell lines modulates the effects on DNA methylation and gene expression. VAT, with its more inflammatory profile, appears to drive epigenetic changes that promote a more aggressive tumor phenotype, particularly in MDA-MB-231. Conversely, SAT may have less impact on the DNA methylome due to its anti-inflammatory properties, especially in the more stable MCF7 cells. Understanding these differences may help develop targeted metabolic or epigenetic therapies for breast cancer subtypes, personalized to the tumor’s molecular profile and influenced by obesity-related factors.

VLCKDs are characterized by low energy density and a reduction in carbohydrate intake leading to nutritional ketosis, with the synthesis of ketone bodies [35, 36]. These types of diets are associated with a reduction in body weight and improvement in quality of life, anthropometric measurements and biochemical parameters [10, 30, 37–39]. Furthermore, this nutritional strategy is capable of reducing visceral fat mass while preserving muscle mass and function, which contributes to reducing inflammation and oxidative stress [10, 40, 41], and with a reversal of the pro-tumoral state due to adipose tissue dysfunction in obesity [42]. After evaluating effect of nutritional ketosis induced by a VLCKD in patients with obesity, and therefore postulating it as an effective treatment for weight loss in previous studies [11, 30, 38, 43], but also for diseases associated with obesity, such as cancer, it was proposed to analyze the role of nutritional ketosis on the methylation of genes related to cancer processes. With GO analysis performed in the current study by a reanalysis of an epigenome-wide study in patients with obesity following a VLCKD [11], it was found that a VLCKD induced methylation changes in 920 genes, of which 18 belonged to cancer-related pathways. Specifically, 5 of these genes were those with the greatest methylation changes, *CCND1*, *GLI2*, *LAMC3*, *CTNNA2 and MAPK10*.

*CCND1* is a critical cell cycle regulator that plays an important role in the expression of several types of cancer. Its overexpression can lead to uncontrolled cell proliferation and cancer progression, which may be relevant in the diagnosis and prognosis of the disease [44–46]. *GLI2*, is a transcription factor that regulates the expression of genes that in turn regulate tumor growth. *GLI2* is a mediator of the Hedgehog signaling pathway that plays critical roles in maintaining tissue homeostasis. Aberrant activation of this signaling pathway is associated with several malignant neoplastic processes [47]. It has recently been associated with worse overall survival in patients with breast cancer [48] and interferes with cell proliferation in colorectal cancer [49]. *LAMC3* is one of the most representative genes of a signature that has been validated to be used as an independent prognostic factor for breast cancer in individualized treatment [50], and its low expression has also been related to a worse prognosis in ovarian cancer [51]. *CTNNA2* is a tumor suppressor gene that is frequently mutated in some types of cancer, such as laryngeal carcinomas, and these mutations are associated with a worse prognosis [52]. Interestingly, the role of *MAPK10* is highlighted, which in addition to belonging to the 5 genes with the greatest changes in methylation in this analysis, when checking the interactions of the 18 methylated genes belonging to cancer-related pathways, *MAPK10* was the one with the greatest number of interactions presented. The production of *MAPK10* is stimulated by ROS accumulation [53] and it regulates cell differentiation, proliferation, apoptosis, and inflammation [54]. Decreased expression levels of *MAPK10* are associated with worse survival and worse prognosis in cancer [53], being found downregulated in breast or ovarian cancer, among others [54]. Likewise, deactivation of *MAPK10* in neurons induces weight gain and alters insulin and leptin signaling, characteristics associated with obesity [55]. In the current study, the methylation levels of MAPK10 was decreased after nutritional ketosis induced by the VLCKD. Overall, these results suggest that ketone bodies could exert a protective effect of carcinogenesis by means of epigenetic mechanisms.

To validate the results observed in blood leukocytes from patients with obesity following a VLCKD, an in vitro approach was performed in tumoral breast cells. The role of obese adipose tissue secreted factors and ketone bodies was evaluated in the context of weight loss treatments. The aim was to analyze their impact on a broad panel of genes related to carcinogenesis. Additionally, the effect of these factors on the tumoral phenotype of breast cells was investigated. In these experiments, the two breast tumor cell lines were exposed to the VAT and SAT secretome from patients with obesity undergoing surgical procedures with bariatric surgery (BS) to observe the effect of obesity on breast cancer, which had previously been evaluated in the cells MCF10A in our research group [5], and to treatments with nutritional ketosis to observe if this exposure produced changes in cell proliferation and subsequently in the expression of genes related to early stages of carcinogenesis. Specifically, the nutritional ketosis was simulated by using β-OHB, the main ketone body produced in ketogenic diets for weight loss such as VLCKD [10]. β-OHB produced a decrease in cell proliferation, as well as a modulation in the expression levels of the studied cancer-related genes in the breast cancer cell lines. On the contrary, exposure to AT secretomes produced an increase in cell proliferation in breast tumor cell lines, which translated into a modulation in the expression of most of the cancer-related genes that was inverse to that observed after the treatment with the ketone body. In this sense, the cellular tumoral phenotype was reduced in cell lines exposed to β-OHB while it was increased after treatment with AT secretomes. These results agree with those recently found in experiments in mice, where it was found that ketogenic diets, in addition to not influencing the metastasis of mammary tumors, ketosis affected the proliferation of tumor cells and also temporarily reduced tumor growth [56]. Along the same lines, a recent study carried out in vitro on tumor cell lines exposed to different ketone bodies showed an inhibition in cell proliferation [27]. On the other hand, excess adipose tissue, mainly central adiposity characterized by excess VAT, as occurs in obesity, contributes to the development, progression and resistance to treatment of some types of cancer [57, 58].

Accordingly with the proliferation results in the cell lines exposed to β-OHB and AT secretomes, a modulation occurred also in the expression levels representative genes-related to cancer. On the one hand, an increase in the expression of tumor suppressor genes has been observed in cells exposed to treatments with β-OHB, in line with what was expected according to previous research that demonstrates that healthy dietary habits and dietary compounds produce positive changes in tumor suppressor genes [59, 60], as well as an increase in expression in genes related to antioxidant protection, some of them with contradictory functions depending on the type of cell or type of cancer [61–63], such as *SIRT1* [63] or *SIRT6* [61, 64, 65]. However, in relation to the expression of genes that are considered tumor promoters, greater variability in the results was observed. Regarding the results on the expression of *DNMTs*, which play an essential role in epigenetic regulation by mediating DNA methylation processes [66], a generalized increase in the expression of *DNMT1* was observed in cells exposed to β-OHB and to AT secretomes. This enzyme plays an important role in maintaining methylation patterns during DNA replication, essential in the preservation of epigenetic memory during cell proliferation. *DNMT1* plays key roles in the regulation of tumor suppressor genes [67]. On the other hand, an increase in the expression levels of *DNMT3A* and *DNMT3B* was also observed in cells exposed to treatments with β-OHB, which catalyze de novo DNA methylation and their overexpression is related to several types of cancer and primary tumors [68, 69], among them they are involved in the oncogenesis and progression of breast cancer [69]. Specifically, *DNMT3B* is associated with abnormal methylation of cancer-related genes [70]. Accordingly, these differential effect of β-OHB or AT secretomes in the cellular tumoral phenotype was correlated with changes in the DNA methylation profile of the cancer-related genes previously identified in leukocytes from patients with obesity treated with an VLCKD to lose weight. This effect was mainly observed in the triple negative receptor breast cancer cell line, MDA-MB-231, whereas the positive estrogen receptor breast cancer cell line, MCF7 did not show changes in the DNA methylation profile. Importantly, differences in DNA methylation depending on treatments in MDA-MB-231 were particularly observed in MAPK10, LAMC3 and GLI2, similar to that observed in blood leukocytes of patients underwent the VLCKD.

The observed differences in the effects on gene expression and DNA methylation between VAT and SAT secretomes, as well as between the two cell lines, can be explained by the intrinsic molecular characteristics of the adipose tissues and the breast cancer subtypes. Visceral adipose tissue (VAT) and subcutaneous adipose tissue (SAT) have distinct secretory profiles [71]. VAT is associated with a more pro-inflammatory phenotype which are known to promote tumor progression and epigenetic changes. In contrast, SAT secretes a lower amount of pro-inflammatory factors and its secretes adiponectin, an anti-inflammatory adipokine with potential tumor-suppressive effects, in higher levels than VAT. The pro-inflammatory secretome of VAT may create a more favorable environment for DNA methylation changes associated with oncogenic pathways [2, 5].This effect could be observed particularly in genes like CCND1 and GLI2. In contrast, the SAT secretome, with its less inflammatory profile, may induce minor or even protective epigenetic modifications. Regarding differences between MDA-MB-231 and MCF7 cell lines, MDA-MB-231 is a triple-negative breast cancer (TNBC) cell line, aggressive and characterized by genomic instability and high epigenetic plasticity [72]. This feature could make it more responsive to external stimuli, such as in this study could be VAT and SAT secretomes or β-OHB treatment. On the other hand, MCF7 is an estrogen receptor-positive (ER+) breast cancer cell line that exhibits more stable epigenomic features and is less prone to rapid DNA methylation changes [73]. As a result, its response to the treatments may be less pronounced or follow different patterns compared to MDA-MB-231. Differences in active signaling pathways and metabolic dependencies between these cell lines likely influence the response to external factors. For instance, the CCND1 gene, involved in cell cycle regulation, and GLI2, a key mediator of the Hedgehog pathway, may show differential methylation due to their roles in the distinct tumorigenic processes of TNBC and ER + subtypes.

As far as we know this is the first study exploring the effect of ketone bodies on methylation profile of breast cancer cell lines. Our findings are consistent with previous studies [74] which also reported a differential response of breast cancer cells to ketone body treatment but in gene expression and metabolism. This agreement reinforces the idea that the metabolic characteristics of each cancer cell line play a crucial role in determining their sensitivity to ketone bodies. MCF7 and MDA-MB-231 vary in molecular features and morphological characteristics. Moreover, they also show discrepancies between activities of cancer-related pathways and bioenergetic differences. MCF7 breast cancer cell lines, in opposition to MDA-MB-231 demonstrates downregulation of JAK/STAT and MAPK transduction pathway and, additionally, NFkappaB and TNFalpha mediated signal transduction pathways [75]. The differential effect of the ketone body on DNA methylation in MCF7 and MDA-MB-231 cell lines may be explained by their distinct metabolic and epigenetic landscapes. MCF7 cells, which are estrogen receptor-positive (ER+), have a different metabolic flexibility compared to the triple-negative MDA-MB-231 cells. It is possible that in MCF7 cells, ketone bodies serve as a source of acetyl-CoA, contributing to increased histone acetylation and subsequent changes in DNA methylation pathways, leading to overall hypermethylation. On the other hand, MDA-MB-231 cells, which exhibit a more glycolytic and aggressive phenotype, may respond differently to ketone bodies, potentially altering one-carbon metabolism and reducing the availability of methyl donors, thus leading to DNA hypomethylation. These differences highlight the complexity of metabolic-epigenetic interactions and suggest that the response to ketone bodies is highly dependent on the specific metabolic context of each cancer cell line.

These results further support the need for a personalized approach when considering metabolic interventions, such as the ketogenic diet, as a complementary strategy in cancer treatment.

## Conclusions

In conclusion, nutritional ketosis induced by VLCKD in the treatment of obesity induces changes in the methylation related to obesity with genes involved in cancer pathways among those epigenetically regulated by this nutritional treatment. This effect was further reflected in vitro in breast cancer cell lines, particularly in triple negative breast cancer cell lines, when treated with ketone bodies and it was observed in different direction from those induced by the VAT secretome related to obesity. The observed epigenetic changes in vitro suggest ketone bodies and obesity-related secretomes influence tumor behavior, possibly through DNA methylation alterations. The distinct responses in MDA-MB-231 and MCF7 indicate that tumor subtype and molecular characteristics play a critical role in determining the epigenetic impact of these treatments. In addition, the differential effects observed between VAT and SAT secretomes may be attributed to the distinct profiles of secreted factors, with a more inflammatory profile characteristic of VAT secretome. Insights into methylation changes may inform strategies for modifying tumor epigenetics via metabolic or dietary interventions. Overall the results of this study, in human and in vitro, highlight the potential role of VLCKD as an adjuvant to anticancer treatment in groups more susceptible to the development of carcinogenesis such as patients with obesity, promoting epigenetic regulation through nutritional ketosis and weight loss that could be involved in the modulation of the tumor development.

## Electronic supplementary material

Below is the link to the electronic supplementary material.

Supplementary Material 1

## Data Availability

No datasets were generated or analysed during the current study.
