# Supplementary material for: Nutritional ketosis modulates the methylation of cancer-related genes in patients with obesity and in breast cancer cells
Source: J Physiol Biochem. 2025 Mar 27;81(2):483–98. doi: 10.1007/s13105-025-01076-9 (PMC12279599; doi:10.1007/s13105-025-01076-9)
Supplement: Supplementary file 1 — Supplementary Material 1 [file 13105_2025_1076_MOESM1_ESM.docx]

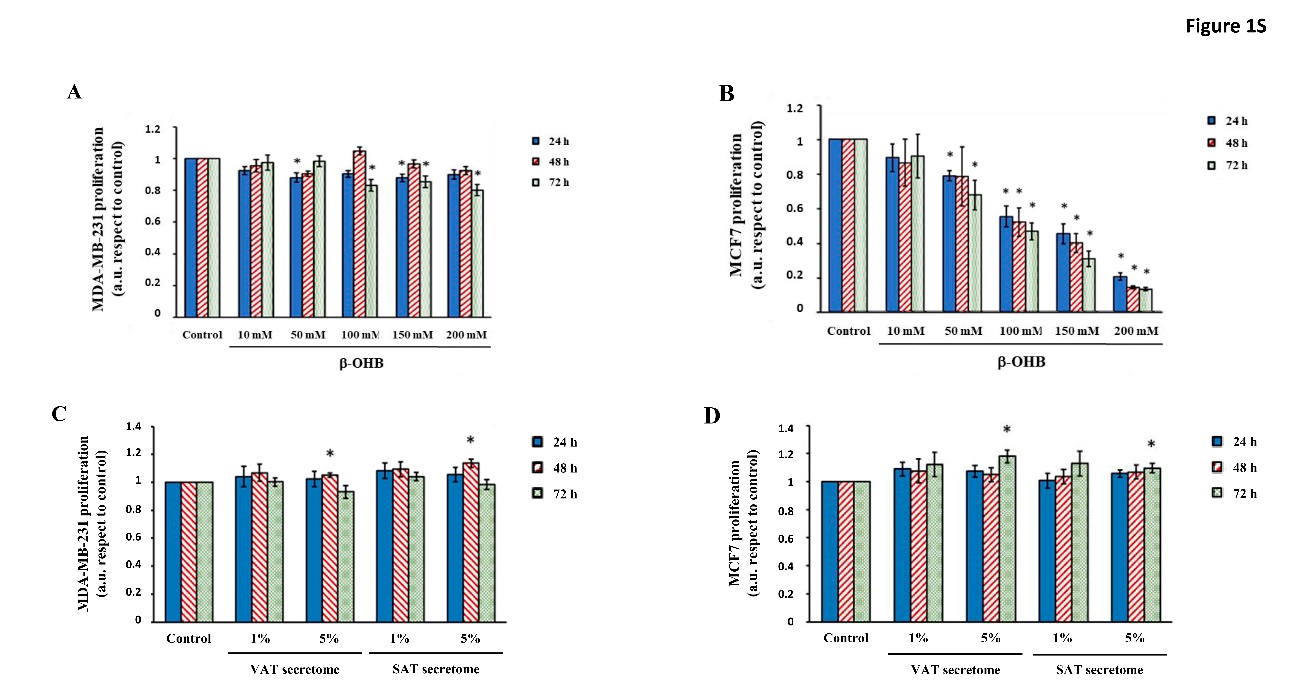


**Figure 1S.** **Cellular proliferation in cell lines. (A) MDA-MB-231 and (B) MCF7 exposed to β-hydroxybutyrate (β-OHB)**. **(B) MDA-MB-231 and (C) MCF7 exposed to adipose tissue secretomes**. Asterisk (*) denotes statistically significant differences (*p* < 0.05) compared to the control evaluated by Student's *t*-test. VAT, visceral adipose tissue; SAT, subcutaneous adipose tissue.


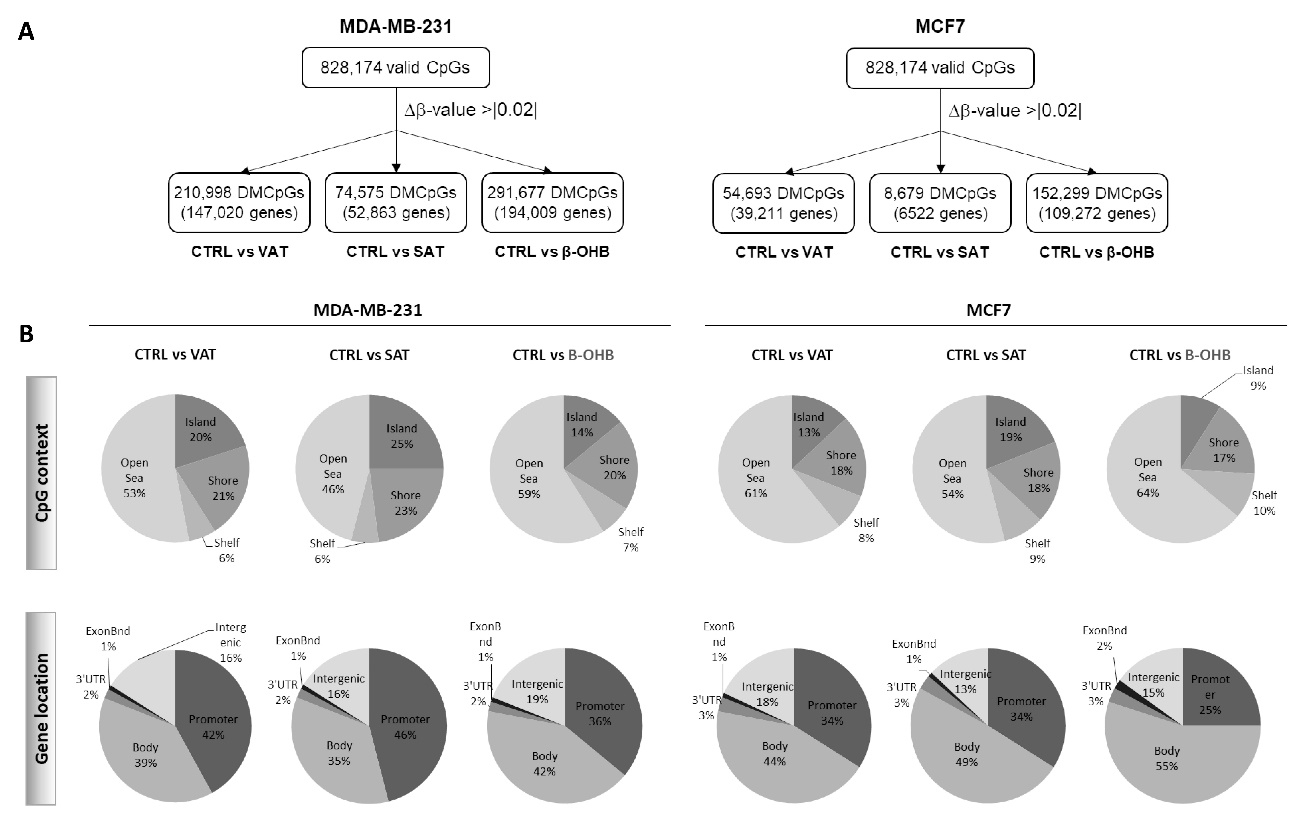


Figure 2S. **Genome-wide DNA methylation analysis of the treated cell lines, MCF7 and MDA-MB-231**. (A) Approach used to identify significant DMCpGs between the treated and the control-untreated cell lines. (B) Description of the DMCpGs found in the treated and control-untreated cell lines according to CpG context and gene. β-OHB, β-hydroxybutyrate; CTR, control; VAT, visceral adipose tissue; SAT, subcutaneous adipose tissue.

**Supplementary Table 1. Probes for RTqPCR (TaqMan®) analysis.**

| Gene | Reference  (Applied Byosistems, EEUU) |
| --- | --- |
| *ALDH3A1* | Hs00964880_m1 |
| *BRCA1* | Hs01556193_m1 |
| *DNMT1* | Hs00945875_m1 |
| *DNMT3A* | Hs01027162_m1 |
| *DNMT3B* | Hs00171876_m1 |
| *GAPDH* | Hs02758991_g1 |
| *GSTM2* | Hs03044640_gH |
| *MYC* | Hs00153408_m1 |
| *PTEN* | Hs02621230_s1 |
| *SIRT1* | Hs01009000_m1 |
| *SIRT3* | Hs00202030_m1 |
| *SIRT6* | Hs00213036_m1 |
| *Survivina/BIRC5* | Hs04194392_s1 |
| *TP53* | Hs01034249_m1 |

*ALDH3A1*, aldehyde dehydrogenase 3A1; *BRCA1*, breast cancer 1; *DNMT*, DNA methyltransferase; *GAPDH*, glyceraldehyde-3-phosphate dehydrogenase; *GSTM2*, glutathione S-transferase Mu 2; *MYC*, myelocytomatosis; *PTEN*, homolog of phosphatase and tensin; *survivin/BIRC5*, baculoviral inhibitor of apoptosis repeat-containing 5; *SIRT*, sirtuin; *TP53*, tumor protein 53.
